# Dose and side effects of sublingual misoprostol for treatment of postpartum hemorrhage: what difference do they make?

**DOI:** 10.1186/1471-2393-12-65

**Published:** 2012-07-07

**Authors:** Wilfrido León, Jill Durocher, Gustavo Barrera, Ernesto Pinto, Beverly Winikoff

**Affiliations:** 1 Hospital Gineco–Obstétrico Isidro Ayora, Quito, Ecuador; 2 Gynuity Health Projects, New York, NY, USA

**Keywords:** misoprostol, postpartum hemorrhage, PPH, fever

## Abstract

**Background:**

Shivering and fever are common side effects of misoprostol. An unexpectedly high rate of fever above 40°C was documented among Ecuadorian women given treatment with 800mcg of sublingual misoprostol to manage postpartum hemorrhage (PPH) (36%). Much lower rates have been reported elsewhere (0-9%).

**Methods:**

From February to July 2010, an open-label pilot study was conducted in Quito, Ecuador to determine whether a lower dose--600mcg sublingual misoprostol--would result in a lower incidence of high fever (≥40°C). Rates of shivering and fever with 600mcg sublingual regimen were compared to previously documented rates in Ecuador following PPH treatment with 800mcg sublingual misoprostol.

**Results:**

The 600mcg dose resulted in a 55% lower rate of high fever compared with the 800mcg regimen (8/50; 16% vs. 58/163; 36%; relative risk 0.45 95% CI 0.23-0.88). Only one woman had severe shivering following the 600mcg dose compared with 19 women in the 800mcg cohort (2% vs. 12%; relative risk 0.17 (0.02-1.25)). No cases of delirium/altered sensorium were reported with the 600mcg dose and women’s assessment of severity/tolerability of shivering and fever was better with the lower dose.

**Conclusions:**

600mcg sublingual misoprostol was found to decrease the occurrence of high fever among Ecuadorian women when given to treat PPH. This study however was not powered to examine the efficacy of this treatment regimen and cannot be recommended at this time. Future research is needed to confirm whether other populations, outside of Quito, Ecuador, experience unusually high rates of elevated body temperature following sublingual administration of misoprostol for treatment of PPH. If indeed similar trends are found elsewhere, larger trials to confirm the efficacy of lower dosages may be justified.

**Trial Registration:**

Clinical trials.gov, Registry No. NCT01080846

## Background

Misoprostol, a prostaglandin E1 derivative, is a safe and effective alternative for managing postpartum hemorrhage (PPH) due to uterine atony [1,2] however, concerns still remain about its side effects profile [3]. The most common side effects associated with the postpartum administration of misoprostol for PPH are shivering and fever [4]. Rates of shivering and fever have been shown to be higher following oral and sublingual routes of administration compared with rectal and vaginal routes [5,6]. Nevertheless, the sublingual administration of misoprostol, due to its rapid onset of action, has been identified as being the most useful application for controlling postpartum bleeding [7].

In several studies, misoprostol has been associated with fever greater than 40.0°C (104°F) when used to manage PPH [1,2,8-13]. Multi-center studies testing an 800mcg regimen of sublingual misoprostol for treatment of PPH reported a higher-than-expected rate of fever above 40°C in one of nine sites (Ecuador: 36%), whereas much lower rates were recorded in the other eight sites, ranging from 0 to 9% [1,2,14]. All of these cases in all sites were transient and resolved within several hours and without complication. Nonetheless, it is unclear why some women developed high body temperature while others did not and why nearly all of the high fevers occurred in Ecuador. Environmental factors, clinical practices and patient characteristics that could possibly contribute to the increased rate of high fevers among Ecuadorian women have been explored, and no patterns or associations have been identified [14]. These unanswered questions pose challenges for providers and policymakers interested in introducing 800mcg sublingual misoprostol as a treatment option in Ecuador. Currently, no data support other routes of administration or lower doses of misoprostol for first-line PPH care. Although there is some evidence that a lower dose would reduce the occurrence of side effects [3,15,16], the effectiveness of a lower dose as first-line PPH treatment has not yet been studied.

To understand better the relationship between misoprostol dose and occurrence of fever, a pilot study was conducted in Quito, Ecuador using a lower dose of sublingual misoprostol for the treatment of PPH. The study aimed to confirm whether a 600mcg dose would reduce the incidence of elevated body temperature (≥40°C) associated with misoprostol. In this manuscript, the side effect profile of the 600mcg sublingual regimen is compared to previously documented rates of shivering and fever using 800mcg sublingual misoprostol in Ecuador. The results from this study help shed light on whether lower doses of misoprostol might be considered and add to the discussion of the possible trade-offs among efficacy, safety (side effects) and acceptability.

## Methods

From February to July 2010, we conducted an open-label study at Hospital Gineco-Obstétrico Isidro Ayora, a large public tertiary care maternity hospital in Quito, Ecuador, to test a 600mcg dose of sublingual misoprostol for the treatment of PPH. The study sought to evaluate the side effect profile and acceptability of sublingual misoprostol (600mcg) as a first-line treatment among women delivering vaginally with suspected uterine atony. The study was approved by the hospital ethics committee and is registered with Clinical trials.gov NCT01080846.

Upon presentation to the labor ward, women were screened for eligibility and informed about the study. Informed consent was obtained for all eligible women and documented via signature or thumb print. Women who had a known allergy to prostaglandin or had a planned cesarean-section were excluded from participation. Consenting women were administered 10 IU oxytocin intramuscularly during the third stage of labor as routine practice at the hospital. Postpartum blood loss was measured following vaginal delivery using a polyurethane receptacle with a calibrated funnel (Brasss-V Drapes®, Excellent Fixable Drapes, India). Socio-demographic and delivery characteristics, pre-delivery hemoglobin and blood loss at one-hour postpartum were documented for all women, regardless of whether they later received treatment for PPH. Women who underwent a cesarean-section or whose postpartum bleeding was not suspected to be due to atonic uterus were later excluded from the study.

Women diagnosed with excessive bleeding that the clinical care team deemed would benefit from uterotonic therapy were enrolled. The treatment regimen consisted of three 200mcg pills of misoprostol given sublingually (under the tongue). Data were collected on the side effects detected by providers or reported by women following treatment administration. Delivery attendants rated the severity (mild, moderate or severe) of any side effect noted and recorded any treatment given to manage it. Body temperature was measured systematically at 60-minutes and 90-minutes for all women given study treatment using an oral mercury thermometer. If the woman’s body temperature measured ≥ 40.0°C at any time, temperature measures were monitored and recorded every half-hour until the fever subsided (below 38.0°C). Management practices for reducing fever included removing blankets from the patient, applying cool compresses, administering oral acetaminophen, and ensuring adequate hydration by mouth or intravenously. All other side effects necessitating treatment were managed according to hospital’s clinical protocol.

Prior to discharge from the hospital, delivery attendants interviewed women about the acceptability of side effects following treatment. Phone follow-up interviews were conducted by study staff one-week after hospital discharge with all women who had experienced high fever to confirm that they had no recurring side effects or health problems that might be related to their treatment.

Women requiring additional care beyond first-line treatment with misoprostol to control postpartum bleeding were provided standard care. Although this study was not powered to confirm the efficacy of the 600mcg sublingual regimen, measures of treatment success were documented to help generate hypotheses for future research addressing the efficacy of the lower dose regimen.

An independent advisory committee was established to review reports of adverse events, provide advice on risk management, and review study findings. Close monitoring of reported side effects continued throughout the study. If at any time the total number of cases of high fever exceeded 15, a stopping rule required that the investigators consult the advisory committee to seek their advice on study continuation and whether a further dose reduction was necessary.

We hypothesized that significantly fewer women would experience high fever following a 600mcg regimen of sublingual misoprostol, compared to a previously documented rate of 36% following 800mcg in Quito, Ecuador [2,14]. Incidence of shivering and fever are related and dose- and route- dependent [3-7]. Previous research on use of misoprostol for PPH has suggested that fever rates are generally halved when misoprostol regimens eliminate one tablet of 200mcg [3,15]. Thus, it was hypothesized that a lower dose of misoprostol would reduce the rate of high fever by 50% in this setting. To detect a one-sided difference of 50% or less, with 80% power at the alpha = 0.05 level, a maximum of 75 cases of PPH was required.

Data were entered and analyzed using the Statistical Package for the Social Sciences, version 15.0 (SPSS, Chicago, IL, USA). Descriptive statistics were calculated for maternal side effects and their severity. Elevated body temperature measuring ≥40.0°C was classified as high fever regardless of its duration. The rate of high fever was then compared to documented rates following use of 800mcg sublingual misoprostol in the previous trial that was conducted at the same hospital in Ecuador [2,14]. Relative risk (RR) and 95% confidence intervals were calculated as appropriate for comparisons between outcomes among Ecuadorian women treated with 600mcg and 800mcg in the present and previous studies, respectively. Importantly, when the previous trial on the 800mcg dose was conducted, systematic provision of oxytocin prophylaxis was not part of the hospital norms; whereas in the present study, administration of oxytocin prophylaxis is now routine. Thus, the comparisons take into account the two different clinical circumstances in which the treatments were offered (600mcg sublingual misoprostol after oxytocin prophylaxis; 800mcg sublingual misoprostol with no previous prophylaxis).

## Results

A total of 764 consenting women were enrolled, of whom 727 had their blood loss measured following vaginal delivery using a calibrated receptacle. Thirty-seven women underwent cesarean-section and were excluded. Among those women who were screened for PPH, 6.9% (50/727) were diagnosed with PPH due to atonic uterus and were administered treatment with 600mcg of sublingual misoprostol. The median blood loss at the time uterotonic treatment was initiated was 850 ml (IQR 750, 1050). Active bleeding was controlled within 20 minutes with misoprostol alone for 82.0% (41/50) of women. Additional uterotonics were administered to nine women, and one woman received a blood transfusion. The median total blood loss for women treated with 600mcg sublingual misoprostol was 1000 ml and the proportion of women with a change in hemoglobin from pre- to post-delivery of 2 g/dL or more was 63.3% (31/49).

Nearly all women given this regimen experienced shivering and fever (Table 1). The incidence of high fever (above 40.0°C) was 16% (8/50) and the highest peak temperature recorded was 40.9°C. Among those women who experienced high fever, average temperatures remained above 40 degrees for less than 1 hour. Only one woman had severe shivering and no cases of delirium or altered sensorium were reported. All other side effects were negligible (Table 1). Baseline characteristics, clinical practices and PPH outcomes (including blood loss measures, hemoglobin levels, and recourse to additional interventions) were similar among women with high fever and those without (data not shown).

**Table 1:** Side effects reported by delivery attendants among Ecuadorian women given PPH treatment with sublingual misoprostol (600mcg and 800mcg) *

	**600 mcg(n = 50)**	**800 mcg(n = 163)**	**RR 95% CI**
Any fever	44 (88.0)	151 (92.6)	0.95 (0.85-1.06)
≥ 40.0°C	8 (16.0)	58 (35.6)	0.45 (0.23-0.88)
Any shivering	48 (96.0)	146 (89.6)	1.07 (0.99-1.16)
Severe shivering	1 (2.0)	19 (11.7)	0.17 (0.02-1.25)
Any fainting	2 (4.0)	4 (2.5)	1.63 (0.31-8.64)
Any nausea	0 (0.0)	8 (4.9)	--
Any vomiting	0 (0.0)	8 (4.9)	--
Any diarrhea	0 (0.0)	2 (1.2)	--
Any delirium or altered sensorium	0 (0.0)	10 (6.1)	--
Any other side effect ^	1 (2.0)	10 (6.1)	0.33 (0.04-2.48)

* Values are given as n (%) unless stated otherwise.

^^^ 600mcg dose -- headache (1); 800mcg dose -- headache (2), thirst (3), pain/cramping (3), excessive sweating (1), Feel cold/teeth chattering (1).

Prior to discharge, women were asked to report any side effects experienced and rate their severity. Among those women confirming provider reports during their exit interview of having had fever, four women characterized the fever as severe or not tolerable (4/31; 12.9%). Eighty-percent (40/50) of women deemed the side effect profile as overall acceptable, 14.0% (7/50) had neutral response to the question, and 6.0%. (3/50) described their side effects as not acceptable.

Women with high fever were contacted by study staff one-week following hospital discharge to interview them about any recurring side effects. Six of the eight women who had high fever were contacted, three of whom reported experiencing mild fever since hospital discharge. The reports of mild fever were not deemed by the study team to require further care. No other problems or concerns were reported by women during their phone follow-up interview.

There were no maternal deaths, no hysterectomies, or other surgeries following first-line treatment with 600mcg sublingual misoprostol. All fifty women were reported to be in good condition upon discharge from the study.

## Discussion

Following treatment with 600mcg sublingual misoprostol for PPH, the incidence of high fever (≥40.0°C) was 55% lower in this study than that recorded in a previous study giving women 800mcg of sublingual misoprostol in Ecuador (relative risk 0.45 95% CI 0.23-0.88) (Table 1). The lower dose did not reduce the overall incidence of shivering and fever following postpartum treatment with sublingual misoprostol in this setting. In both studies, approximately nine out of ten women experienced shivering and fever irrespective of the dose received (Table 1). Nevertheless, perceived tolerability/severity of fever was significantly improved following the lower treatment dose. Only four women treated with 600mcg characterized the fever as severe or not tolerable (4/31; 12.9%). In comparison, 43.6% (44/101) of women in the 800mcg study reporting fever in their exit interview described this side effect as severe or not tolerable (relative risk 0.30 95% CI 0.12-0.76). There were no reports of delirium or altered sensorium among those treated with 600mcg sublingual misoprostol; whereas eleven (11/163; 6.7%) women reported this effect when given 800mcg sublingual misoprostol to treat hemorrhage.

**Figure 1:**
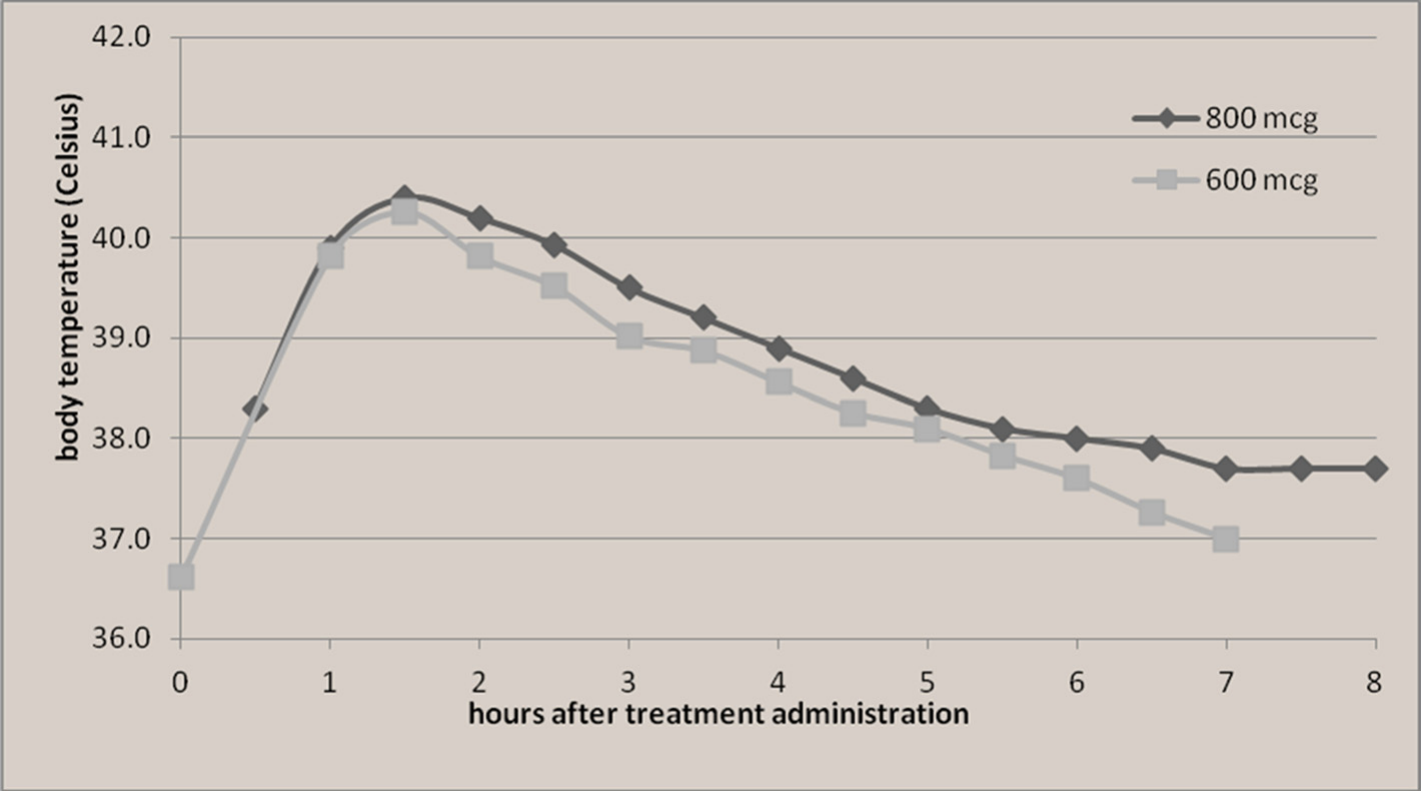
Average temperature trends for cases with high fever following 600 mcg (n=8) and 800mcg (n=58) doses of misoprostol for PPH treatment.

These results provide further evidence of the dose-dependent nature of misoprostol-induced side effects. Shivering and fever were commonly experienced by Ecuadorian women following both the 600mcg and 800mcg doses studied, yet the intensity of these effects appears to be reduced with the lower dose. For instance, the lower dose was associated with a lower peak temperature (40.9°C vs. 41.8°C in the 800mcg study) and, on average, a 30-minute shorter duration of temperature above 40.0°C, compared with those treated with 800mcg sublingual misoprostol in the previous study (Figure 1). Only one woman experienced severe shivering in the 600mcg cohort compared with 19 women treated with 800mcg given sublingually (2% vs. 12%; relative risk 0.17 (0.02-1.25)) (Table 1). A similar pattern was noted in one study that compared the side effect profiles of 200mcg, 400mcg, and 600mcg regimens of sublingual misoprostol for the management of PPH among Libyan women [17]. In all three treatment groups, the majority of women had shivering (75%-100%) and fever (83%-100%) confirmed by systematic temperature measurement, yet only 8% in the 200mcg and 400mcg groups had fever above 39.0°C, compared with 45% given 600mcg sublingually. There were no cases of fever above 40.0°C in Libya [17].

Although the lower dose of sublingual misoprostol was associated with a reduction in the rate of high fever in Ecuador, the proportion of Ecuadorian women experiencing high fever (16%) is considerably higher than rates documented in studies using the same regimen for PPH indications. In one large multi-country study, body temperature following adjunct PPH treatment with 600mcg of sublingual misoprostol was systematically measured, confirming a rate of high fever of 7% (48/704) [13]. In Pakistan, a study of the adjunct use of 600mcg sublingual misoprostol for treatment of PPH documented a 3% rate of high fever (1/29) [12]. Six other PPH prevention studies utilizing 600mcg sublingual misoprostol report no cases of fever above 40.0°C among a total of 835 women given this regimen [16-21]. Clinical practices and patient characteristics of Ecuadorian women treated with 600mcg sublingual misoprostol were analyzed to explore whether there were any patterns or associations with the occurrence of high fever, and none were identified. Thus, we have hypothesized that the thermoregulatory response to misoprostol is based on genetic factors, which would explain the variable frequency of fever/high fever among different populations. Pharmaco-genetic analyses are underway to determine whether genetic variability in genes encoding enzymes responsible for metabolism and transport of misoprostol can explain why Ecuadorian women experience higher rates of elevated temperature (personal communication, Ana Alfirevic, The University of Liverpool).

The findings from this pilot study are limited due to the small sample size. The study was only powered to detect a difference in the rate of high fever between the two treatment regimens and the sample size was not sufficient to study the efficacy of 600mcg dose. Furthermore, due to the infrequency of PPH events following routine use of oxytocin prophlaxis during the timeframe of the pilot study, the original calculated sample size of 75 PPH cases was not met. An additional limitation relates to the study design as the results from the 600mcg dose were compared with historical cohort data from a previous study conducted in Ecuador. The trend towards a reduction in high fevers may have been influenced by other factors, such as the provision of oxytocin prophylactically in the present study, which was not routinely practiced in the study by Winikoff et al. Nevertheless, the participant characteristics were similar between the two studies (Table 2), and the reduction in fever, as well as the temperature curve following administration of 600mcg, is consistent with other studies that have compared misoprostol doses and systematically measured body temperature [5,17,22]. The findings from this study may also have limited generalisability, as it is not yet known whether women in other settings (for example, other South American populations) will also experience similar rates of high fever following postpartum administration of sublingual misoprostol in dosages of 600 or 800mcg.

**Table 2:** Participant characteristics and PPH outcomes among Ecuadorian women given PPH treatment with sublingual misoprostol (600mcg and 800mcg)*

	**600 mcg(n = 50)**	**800 mcg(n = 163)**
**Baseline characteristics**		
Age (yr) mean ± sd,	23 ± 6	24 ± 6
Range	14 – 41	14 – 42
Currently married	39 (78.0)	140 (85.3)
No education	0 (0.0)	0 (0.0)
Primary	11 (22.0)	50 (30.7)
Secondary	29 (58.0)	96 (58.9)
University or higher	10 (20.0)	14 (8.6)
0	27 (54.0)	65 (39.9)
1–3	21 (42.0)	86 (52.8)
4+	2 (4.0)	12 (7.3)
Pre-delivery Hb mean ± sd,	13.4 ± 1.4	13.5 ± 1.3
Range	10.1 – 18.1	10.3 – 16.9
Pre–term (less than 37)	2 (4.0)	6 (3.7)
Term (37·0 – 40·9)	42 (84.0)	137 (84.0)
Post–term (41 or more)	6 (12.0)	20 (12.3)
Multiple pregnancy	0 (0)	0 (0)
Known previous PPH	1 (2.0)	8 (4.9)
**Delivery characteristics**		
Labor induced/augmented	34 (68.0)	--
Oxytocin given in 3rd stage of labor	50 (100.0)	--
Controlled cord traction ^+^	45 (90.0)	114 (69.9)
Uterine massage ^+^	50 (100.0)	131 (80.4)
**PPH outcomes**		
Blood loss (mL) at time of treatment median (IQR)	850 (750,1050)	850 (750, 1000)
Total blood loss (mL) median (IQR)^+^	1000 (850, 1200)	1150 (950, 1400)
Blood loss ≥500 mL post-treatment	5 (10.0)	29 (17.8)
Blood loss ≥1000 mL post-treatment	0 (0.0)	3 (6.0)
Post-treatment Hb median IQR ^^+^	10.8 (9.9, 11.4)	9.9 (8.9, 11.0)
Difference between pre/post Hb median IQR^^+^	2.6 (1.4, 4.0)	3.5 (2.6, 4.8)
Drop in Hb ≥ 2 g/dL ^^+^	31 (63.3)	131 (85.6)
Additional uterotonics ^+^	9 (18.0)	12 (7.4)
Blood transfusion	1 (2.0)	10 (6.1)
Exploration under anesthesia	9 (18.0)	16 (9.8)

* Values are given as n (%) unless stated otherwise.

^ Post-delivery hemoglobins available for 49 women in the 600mcg study and 153 women in 800mcg study.

+ *P* ≤ 0.05.

While this study was not designed to evaluate the effectiveness of the 600mcg dose, we did document outcomes related to treatment success, including time to bleeding cessation, measured blood loss, change in hemoglobin levels measured pre- and post-delivery, and use of additional interventions (Table 2). First-line treatment with 600mcg sublingual misoprostol alone controlled PPH within 20 minutes for 82% of women. In comparison, two large multi-country randomized controlled trials testing an 800mcg regimen of sublingual misoprostol in the presence and absence of oxytocin prophylaxis confirmed rates of efficacy of 89.2% (363/407) and 90.2% (440/488), respectively [1,2]. Direct comparisons of PPH outcomes from the two Ecuador cohorts show that women bled less and had better hemoglobin levels after administration of 600mcg misoprostol (Table 2). Importantly, the outcomes in the present study are likely the result of the package of PPH care that women received -- a prophylactic regimen of 10 IU oxytocin followed by treatment with 600mcg sublingual misoprostol vs. treatment alone in the previous study. Routine use of oxytocin during the third stage of labor also significantly reduced the rate of PPH diagnosis by 75% in this setting (no prophylaxis: 29% (325/1624) vs. prophylaxis: 7% (50/727)); thus reducing the number of women requiring treatment with oxytocics and their exposure to any side effects from these drugs.

Although some evidence suggests that lower doses of misoprostol may have the same effect on the uterus compared with higher doses [17], no large randomized controlled trials have been conducted confirming the efficacy of lower doses for treating PPH. Indeed, a comparative trial of 600 vs. 800mcg may not be practical due to the large sample size and the financial resources required to confirm non-inferiority of a lower dose. Furthermore, the 82% rate of effectiveness, defined as blood loss controlled within 20 minutes with misoprostol treatment alone, documented in the present study suggests a possible loss in efficacy with the lower dose -- a trade-off that may not be acceptable to providers when treating a life-threatening obstetric condition.

## Conclusions

First-line treatment with 600mcg of sublingual misoprostol, preceded by the intramuscular provision of 10 IU of oxytocin, works well in controlling hemorrhage and minimizing the occurrence of high fever among Ecuadorian women. The lower misoprostol dose offers a potential solution for treating PPH in communities known to be more susceptible to higher rates of misoprostol’s thermoregulatory effects. However, these findings do not provide conclusive evidence of misoprostol’s efficacy using this lower dose. Moreover, there is no evidence that this dosage alone in the absence of oxytocin prophylaxis would be an effective intervention for controlling PPH and avoiding side effects. Until additional information becomes available on the effectiveness of lower dosages, the evidence-based 800mcg sublingual regimen is recommended, particularly in settings where the provision of intravenous oxytocin is not feasible [1,2]. Operations research utilizing the 800mcg sublingual regimen in other settings is recommended to confirm the safety and acceptability of this approach to manage cases of PPH. If indeed unusually high rates of elevated body temperature are documented among other populations, future research on the efficacy of lower dosages for treating PPH may be justified.

## Abbreviation

PPH, Postpartum hemorrhage.

## Competing interests

The authors declare that they have no competing interests.

## Authors’ contributions

WL: contributed to the conception of the trial, participated in its implementation and interpretation of the trial findings, and helped draft the manuscript. JD: contributed to the conception of the trial, provided clinical monitoring, analyzed the study findings and drafted the manuscript. GB: participated in the study implementation and interpretation of results. EP: participated in the study implementation, managed the data collected, and participated in the interpretation of result. BW: contributed to the conception of the trial, helped interpret data and draft the manuscript. All authors read and approved the final manuscript.

## Pre-publication history

The pre-publication history for this paper can be accessed here:

http://www.biomedcentral.com/1471-2393/12/65/prepub
